# Comparative efficacy of various non-pharmacological therapies on cognitive function in patients with mild cognitive impairment or dementia: A network meta-analysis

**DOI:** 10.1097/MD.0000000000050854

**Published:** 2026-09-25

**Authors:** Hongxia Zhou, Ting Ma, Lingling Yang, Xian Sun, Jiafei Yang

**Affiliations:** a Department of Epidemiology and Health Statistics, School of Public Health, Ningxia Medical University, Yinchuan, China; b Key Laboratory of Environmental Factors and Chronic Disease Control, Ningxia Medical University, Yinchuan, China.

**Keywords:** cognitive function, dementia, mild cognitive impairment, network meta-analysis, non-pharmacological therapy

## Abstract

**Background:**

Evidence directly comparing the cognitive improvement effects of non-pharmacological therapies in patients with mild cognitive impairment or dementia is limited. Our objective was to evaluate the efficacy of various non-pharmacological therapies on cognitive function through a network meta-analysis.

**Methods:**

PubMed, Embase, Cochrane Library, China Biology Medicine Database, China National Knowledge Infrastructure, Wanfang Database, and Web of Science were searched from database inception until October 2025. Two researchers independently screened studies, extracted data, and assessed bias risk.

**Results:**

Twenty-four randomized controlled trials (2082 patients) compared multidomain intervention (MI), music therapy (MT), cognitive rehabilitation (CR), physical exercise (PE), cognitive training (CT), and health education (HE). For Mini-Mental State Examination scores, CT (standardized mean difference [SMD] = 1.43, 95% confidence interval [CI] = 0.20–2.65) and HE (SMD = 2.02, 95% CI = 0.05–4.00) showed statistically significant improvements versus control (*P* < .05). Exploratory P-score ranking yielded values of 0.26 (MI), 0.39 (MT), 0.49 (CR), 0.59 (PE), 0.73 (CT), and 0.86 (HE), suggesting HE and CT may carry higher efficacy probability within this network. Regarding the Montreal Cognitive Assessment scores, PE (SMD = 2.26, 95% CI = 0.95–3.57), HE (SMD = 2.43, 95% CI = 0.43–4.43), and CT (SMD = 2.96, 95% CI = 1.57–4.35) showed statistically significant improvements compared to the control group (*P* < .05). Exploratory P-score ranking gave values of 0.07 (MI), 0.46 (MT), 0.69 (PE), 0.73 (HE), and 0.89 (CT), indicating CT had the highest preliminary ranking probability.

**Conclusion:**

The results of this study indicate that PE, CT, and HE show good potential for improving cognitive function, but the screening results of different assessment tools show a certain degree of variability. MT, CR, and MI did not show significant efficacy. More randomized controlled trials may need to be included in the future.

## 1. Introduction

Cognitive impairment is a multifactorial syndrome involving genetic, neurodegenerative, vascular, and metabolic mechanisms, manifesting as a clinically significant decline in memory, executive function, or language, and interfering with daily living.^[1,2]^ The spectrum ranges from mild cognitive impairment (MCI) to dementia. MCI represents a transitional stage between normal aging and dementia, with approximately 10% to 15% of patients progressing to dementia annually.^[3]^ Importantly, interventions during the MCI stage may mitigate cognitive decline and potentially delay or prevent dementia onset.^[4]^

Currently, interventions for MCI or dementia fall into 2 categories: pharmacologic and non-pharmacologic. Pharmacologic options, such as cholinesterase inhibitors and N-Methyl-D-Aspartate receptor antagonists, have limited efficacy and may cause gastrointestinal and cardiovascular side effects that restrict long-term adherence.^[5,6]^ In contrast, non-pharmacological therapies (NPTs) – including cognitive training (CT), physical exercise (PE), cognitive rehabilitation (CR), music therapy (MT), and psychological interventions – are safer and have attracted increasing research interest, with studies demonstrating their potential to slow cognitive decline.^[7,8]^ However, direct comparisons among different NPTs are scarce, and their relative effectiveness remains unclear.^[9]^

Given the lack of high-quality evidence directly comparing NPTs for cognitive improvement in MCI or dementia, we conducted a network meta-analysis (NMA) to compare the effects of various NPTs on cognitive function in this population, aiming to inform clinical decision-making.

## 2. Materials and methods

### 2.1. Literature search strategies

The computer searched PubMed, Embase, Cochrane Library, Chinese Biomedical Literature Database, China National Knowledge Infrastructure Database, Wan Fang Database, and Web of Science databases, from their inception to October 2025. The specific search strategy was shown in Table S1, Supplemental Digital Content 1.

### 2.2. Inclusion and exclusion criteria

The detailed inclusion criteria were as follows: participants must be diagnosed with MCI or dementia; the interventions must include CT, CR, PE, MT, health education (HE), or multidomain intervention (MI); the control group must receive no intervention, routine care, HE, or other forms of nonspecific interventions; the study must report at least one of the following outcomes: Mini-Mental State Examination (MMSE) scores or Montreal Cognitive Assessment (MoCA) scores; and the study design must be a randomized controlled trial (RCT).

The exclusion criteria applied in our study selection process included: research involving participants with comorbid neurological conditions affecting cognition (e.g., epilepsy or multiple sclerosis); redundant or overlapping publications; and studies only available as conference abstracts without full-text papers.

### 2.3. Literature selection and data extraction

The study selection process employed a dual-reviewer system to ensure methodological rigor. Two independent researchers conducted parallel literature screening and data extraction, followed by cross-verification of collected information. Any discrepancies between reviewers were resolved through consensus discussions or, when required, arbitration by a senior investigator. Our screening protocol involved initial title evaluation, followed by abstract assessment of potentially relevant studies, culminating in full-text review of selected articles. For studies with incomplete or ambiguous data, we proactively contacted corresponding authors via electronic correspondence or telephone to obtain clarification. The extracted dataset encompassed: study identification parameters (title, primary investigator, geographic origin); demographic and intervention characteristics; methodological quality indicators; and quantitative outcome measures.

### 2.4. Risk of bias assessment

The methodological quality evaluation was conducted independently by 2 investigators utilizing the standardized risk-of-bias assessment framework outlined in the Cochrane Handbook (v5.1.0; Cochrane Collaboration). Following individual evaluations, the reviewers compared their assessments and reconciled any inconsistencies through structured discussion. When consensus could not be reached, a third senior researcher was consulted for final determination. The appraisal examined 7 critical domains, with each criterion rated as demonstrating either “low,” “high,” or “unclear” potential for bias.

### 2.5. Statistical analyses

Our statistical approach commenced with assessing clinical and methodological homogeneity across studies through similarity hypothesis testing.^[10]^ Treatment efficacy was quantified using frequentist statistical methods. We systematically examined aggregated study data and participant demographics, while heterogeneity was measured via *I*^2^ statistics.^[11]^ The *I*^2^ metric (range: 0%–100%) quantified between-study variation, with thresholds of 25%, 50%, and 75% representing low, moderate, and substantial heterogeneity respectively. Following NMA, we employed funnel plot visualization to detect potential publication bias. A random-effects model was selected for all analyses as it provides more conservative variance estimates in the presence of inter-study variability.^[12]^

The network geometry was visualized using R statistical software (v4.0.2; R Core Team), with distinct therapeutic approaches depicted as nodal points and direct comparative relationships shown as connecting edges. To evaluate coherence between direct and indirect evidence, we performed global inconsistency tests and node-splitting analyses, adopting a consistency model when *P* values exceeded the .05 threshold.^[13]^ Treatment hierarchies were established using P-scores (scale: 0–1), where elevated values corresponded to greater therapeutic efficacy.^[14]^ Potential publication bias was assessed graphically via Review Manager (v5.4.1; Cochrane Collaboration). Furthermore, we executed stratified analyses examining geographical and temporal variations.

## 3. Results

### 3.1. Search process

Figure 1 illustrates the literature screening process. In this study, a total of 19,045 articles were retrieved. After excluding 3110 duplicate articles and 15,819 irrelevant articles, 116 articles remained for full-text review. Ultimately, 24 articles met the inclusion criteria and were included in our NMA (MI = 6 studies, MT = 3 studies, CR = 1 study, PE = 5 studies, CT = 7 studies, and HE = 2 studies). The research team unanimously agreed upon all methodological approaches for study selection and quality assessment.

**Figure 1.**
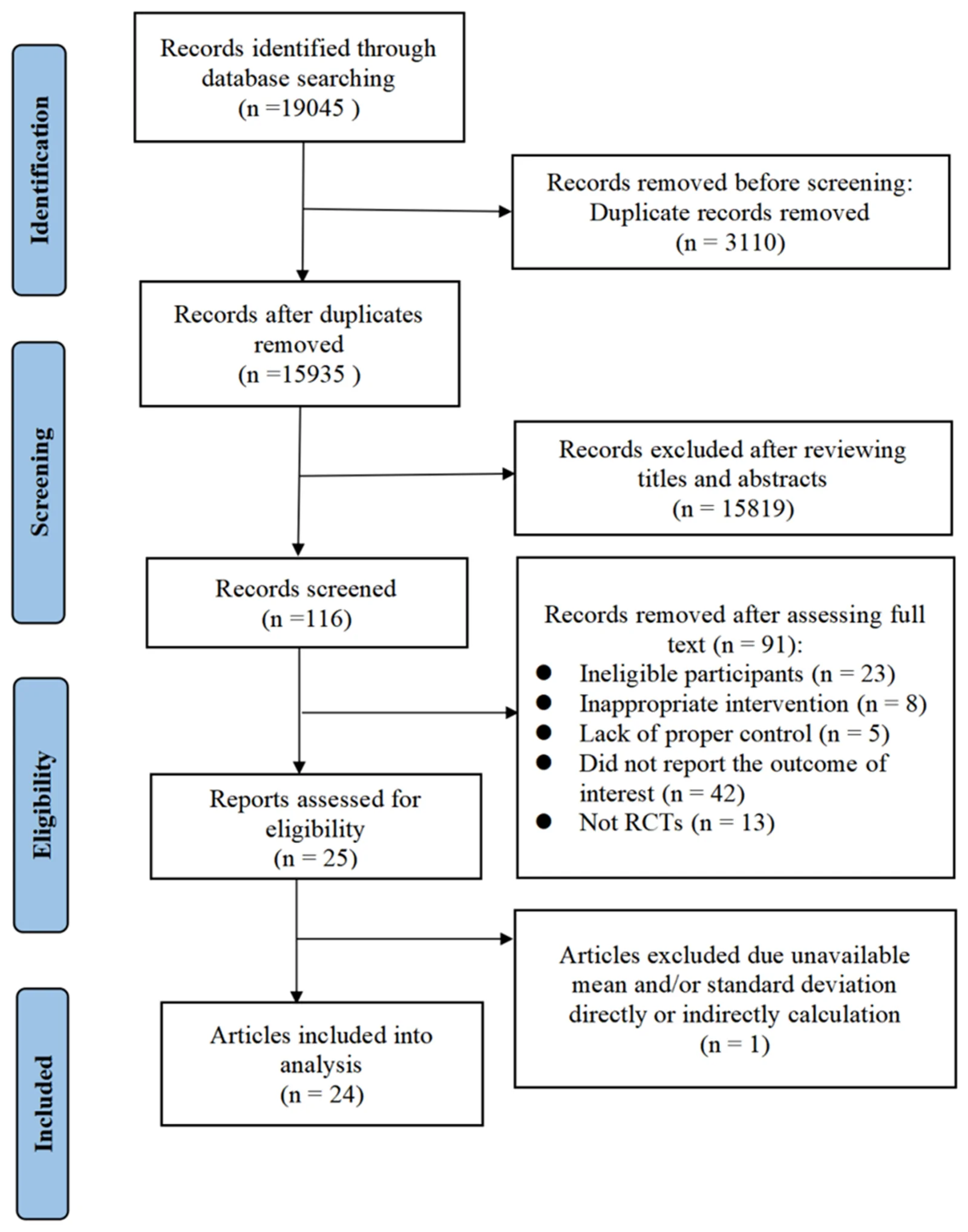
Literature review flowchart. RCT = randomized controlled trial.

### 3.2. Baseline characteristics and bias risk assessment chart

The basic characteristics of the included studies are presented in Table 1.^[15–38]^ A total of 2082 participants were included (intervention: 1087, control: 995). The mean age of the participants ranged from 60 to 87.63 years. Our analysis incorporated 24 eligible studies. Of these, 17 documented specific randomization procedures (e.g., random number tables, sealed envelopes), whereas 7 simply stated randomization without methodological details. Allocation concealment mechanisms were explicitly described in 8 studies. Due to the inherent nature of NPTs, achieving double-blinding proved methodologically challenging – only 2 studies reported such procedures. Fourteen studies implemented blinded outcome assessment, while 4 failed to report attrition rates or reasons. Overall, the included studies demonstrated minimal risks of selective reporting and other biases, with comprehensive quality assessment findings illustrated in Figure 2.

**Table 1:** Characteristics of included studies.

Study	Country	Dementia/MCI	Sample size	Gender (M/F)	Mean age	Intervention	Outcome
Wen et al^[15]^	China	MCI	CT: 37	CT: 21/16	CT: 66.78 ± 8.30	CT	MoCA
			CON: 81	CON: 49/32	CON: 65.75 ± 9.41		MMSE
Diaz et al^[16]^	Spain	Dementia	CT: 36	CT: 13/23	CT: 73.64 ± 6.56	CT	MMSE
			CON: 26	CON: 4/22	CON: 76.23 ± 6.56		
Carcelen et al^[17]^	Spain	MCI	CT: 36	CT: 25/11	CT: 75.36 ± 3.74	CT	MoCA
			CON: 35	CON: 23/12	CON: 74.80 ± 3.92		MMSE
Schmidt et al^[18]^	Germany	MCI	CT: 28	CT: 21/7	CT: 67.18 ± 7.01	CT	MoCA
			CON: 26	CON: 15/11	CON: 67.50 ± 8.71		
Xue et al^[19]^	China	MCI	CT: 36	CT: 11/25	CT: 75.42 ± 4.57	CT	MoCA
			CON: 36	CON: 13/23	CON: 73.44 ± 4.88		
Trebbastoni et al^[20]^	Italy	Dementia	CT: 45	CT: 19/26	CT: 74.26 ± 6.97	CT	MMSE
			CON: 85	CON: 33/52	CON: 76.01 ± 6.46		
Hwang et al^[21]^	Korea	MCI	CT: 5	CT: 1/4	CT: 63.40 ± 9.40	CT	MMSE
			CON: 5	CON: 2/3	CON: 67.20 ± 8.80		
Greenaway et al^[22]^	US	MCI	CR: 20	CR: 8/12	CR: 72.70 ± 6.90	CR	MMSE
			CON: 20	CON: 8/12	CON: 72.30 ± 7.90		
Zhang et al^[23]^	China	MCI	PE: 25	PE: 6/19	PE: 70.08 ± 5.19	PE	MoCA
			CON: 25	CON: 7/18	CON: 69.88 ± 5.31		MMSE
Li et al^[24]^	China	MCI	PE: 42	PE: 15/27	PE: 60.0–80.0	PE	MoCA
			CON: 42	CON: 18/24	CON: 60.0–80.0		MMSE
Song et al^[25]^	China	MCI	PE: 60	PE: 12/48	PE: 76.22 ± 5.76	PE	MoCA
			CON: 60	CON: 18/2	CON: 75.33 ± 6.78		
Lamb et al^[26]^	UK	Dementia	PE: 329	PE: 195/134	PE: 76.90 ± 7.90	PE	MMSE
			CON: 165	CON: 106/59	CON: 78.40 ± 7.60		
Henskens et al^[27]^	Netherlands	Dementia	PE: 22	PE: 5/17	PE: 85.14 ± 4.64	PE	MMSE
			CON: 22	CON: 5/17	CON: 84.73 ± 4.55		
Mangiacotti et al^[28]^	UK	MCI	MT: 23	MT: 3/20	MT: 84.86 ± 7.37	MT	MoCA
			CON: 19	CON: 4/15	CON: 87.63 ± 7.58		
Xue et al^[29]^	China	MCI	MT: 40	MT: 10/30	MT: 75.43 ± 4.75	MT	MoCA
			CON: 40	CON: 8/32	CON: 74.43 ± 4.47		
Biasutti et al^[30]^	Italy	MCI	MT: 25	MT: 8/17	MT: 83.79 ± 7.31	MT	MMSE
			CON: 26	CON: 11/15	CON: 84.42 ± 7.04		
Young et al^[31]^	China	MCI	HE: 18	HE: 4/14	HE: 75.50 ± 6.45	HE	MoCA
			CON: 20	CON: 1/19	CON: 74.50 ± 5.71		
Casemiro et al^[32]^	Brazil	MCI	HE: 10	HE: 3/7	HE: 68.0 ± 6.05	HE	MMSE
			CON: 12	CON: 3/9	CON: 77.3 ± 6.31		
Wang et al^[33]^	China	MCI	MI: 62	MI: 23/39	MI: 66.39 ± 4.24	MI	MMSE
			CON: 61	CON: 25/36	CON: 67.82 ± 4.81		
Fan et al^[34]^	China	MCI	MI: 59	MI: 23/36	MI: 66.30 ± 4.40	MI	MoCA
			CON: 57	CON: 22/35	CON: 67.80 ± 5.10		MMSE
Sung et al^[35]^	China	Dementia	MI: 36	MI: 5/31	MI: 83.50 ± 6.36	MI	MMSE
			CON: 36	CON: 12/24	CON: 81.0 ± 6.41		
Park et al^[36]^	Korea	MCI	MI: 25	MI: 8/17	MI: 70.55 ± 6.46	MI	MMSE
			CON: 24	CON: 7/17	CON: 72.76 ± 5.37		
Han et al^[37]^	Korea	Dementia	MI: 32	MI: 10/22	MI: 75.63 ± 6.30	MI	MMSE
			CON: 32	CON: 14/18	CON: 76.81 ± 5.36		
Hagovská et al^[38]^	Slovak Republic	MCI	MI: 40	MI: 22/18	MI: 68.0 ± 4.40	MI	MMSE
			CON: 40	CON: 19/21	CON: 65.9 ± 6.20		

CON = control, CR = cognitive rehabilitation, CT = cognitive training, HE = health education, MCI = mild cognitive impairment, MI = multidomain interventions, MMSE = Mini-Mental State Examination, MoCA = The Montreal Cognitive Assessment, MT = musical therapy, PE = physical exercise.

**Figure 2.**
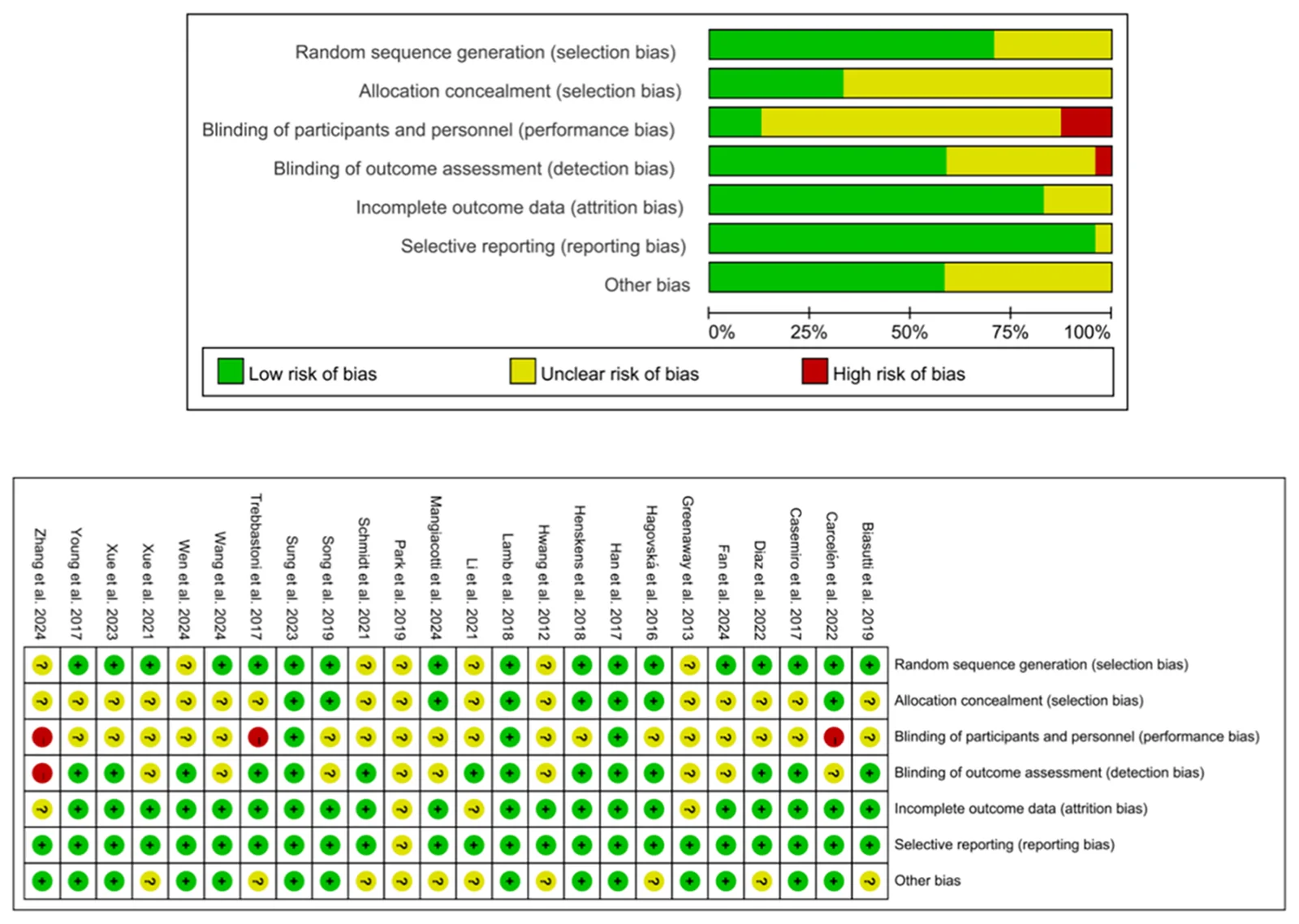
Risk of bias assessment graph.

### 3.3. Network meta-analysis

#### 3.3.1. Evidence network

Network evidence for different interventions is illustrated in Figure 3. In the network diagram, the proportional scaling of nodal circles corresponds to intervention sample sizes, while connecting line weights reflect the quantity of comparative studies between paired interventions. A total of 18 studies reported MMSE scores for a total of 6 interventions, forming 4 direct comparisons and 1 closed loop. In addition, 11 studies reported MoCA scores for a total of 5 interventions, forming 2 closed loops. This suggested the existence of both direct and indirect comparisons.

**Figure 3.**
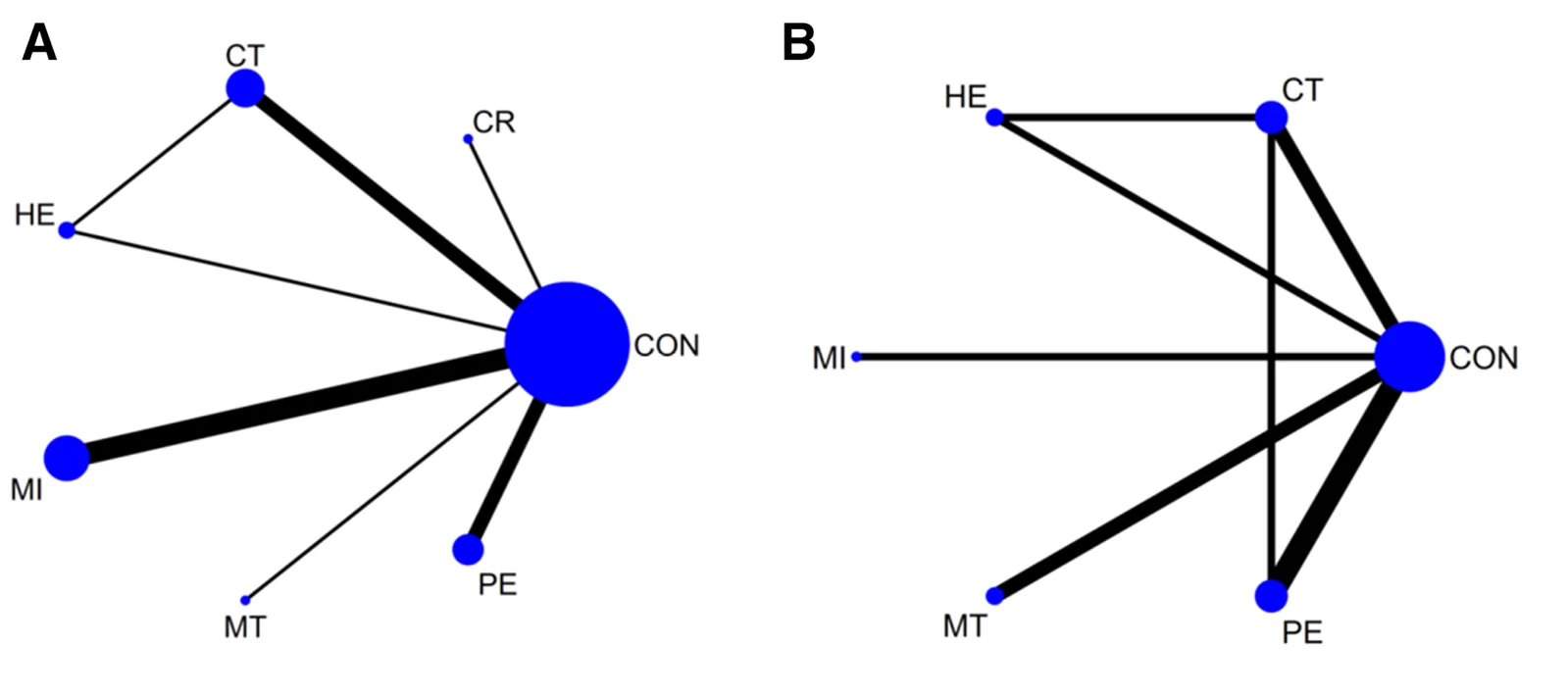
Treatment network graphs for (A) MMSE and (B) MoCA cognitive assessments. Node size corresponds to the total sample size of each intervention; line width represents the number of direct comparative RCTs. CON = control, CR = cognitive rehabilitation, CT = cognitive training, HE = health education, MI = multidomain intervention, MMSE = Mini-Mental State Examination, MoCA = Montreal Cognitive Assessment, MT = music therapy, PE = physical exercise, RCT = randomized controlled trial.

#### 3.3.2. MMSE scores

Preliminary NMA of the included studies demonstrated *I*^2^ = 88.5%. The symmetrical distribution of the funnel plot suggested the absence of significant publication bias in this study (Fig. S1, Supplemental Digital Content 2). In this paper, we evaluated coherence between direct and indirect evidence through comprehensive inconsistency analyses. The global inconsistency test yielded a nonsignificant *P* value (.1511), while node-splitting analyses for specific comparisons showed similarly nonsignificant results: CT versus control (CON; *P* = .1504), HE versus CON (*P* = .1500), and CT versus HE (*P* = .1504). These findings collectively support the adequacy of the consistency model (Table S4, Supplemental Digital Content 3 and Fig. S2, Supplemental Digital Content 4).

Our analysis identified several NPTs that showed statistically significant improvements relative to control conditions. The magnitude of these therapeutic effects was quantified as follows: CT (standardized mean difference [SMD] = 1.43, 95% CI = 0.20–2.65) and HE (SMD = 2.02, 95% CI = 0.05–4.00). In contrast, no significant therapeutic benefits were observed for MI (SMD = 0.06, 95% CI = −0.96 to 1.08), MT (SMD = 0.35, 95% CI = −0.91 to 2.61), CR (SMD = 0.70, 95% CI = −2.05 to 3.45), or PE (SMD = 0.96, 95% CI = −0.42 to 2.34). The exploratory P-scores for CT and HE were 0.73 and 0.86, respectively (Table 2 and Fig. 4). In addition, a comprehensive comparison of multiple NPTs used for 2 different diseases was conducted, with the MMSE serving as the analytical tool. As shown in Figures S5 and S6, Supplemental Digital Content 5, compared with the control group, PE (SMD = 1.51, 95% CI = 0.31–2.89), CT (SMD = 1.86, 95% CI = 0.71–3.01), and HE (SMD = 2.11, 95% CI = 0.55–3.67) significantly improved cognitive function in patients with MCI.

**Table 2:** The influence of NPTs on cognitive performance was determined through standardized cognitive assessments (MMSE).

Group	CON	CR	CT	HE	MI	MT	PE
CON	–	–	–	–	–	–	–
CR	−0.70 (−3.45, 2.05)	–	–	–	–	–	–
CT	−**1.43** (−**2.65, −0.20**)	−0.73 (−3.74, 2.28)	–	–	–	–	–
HE	−**2.02** (−**4.00, −0.05**)	−1.32 (−4.71, 2.06)	−0.59 (−2.46, 1.27)	–	–	–	–
MI	−0.06 (−1.08, 0.96)	0.64 (−2.29, 3.57)	1.37 (−0.23, 2.96)	1.96 (−0.26, 4.19)	–	–	–
MT	−0.35 (−2.61, 1.91)	0.35 (−3.21, 3.91)	1.08 (−1.49, 3.65)	1.67 (−1.33, 4.67)	−0.29 (−2.77, 2.19)	–	–
PE	−0.96 (−2.34, 0.42)	−0.26 (−3.33, 2.82)	0.47 (−1.37, 2.31)	1.06 (−1.35, 3.47)	−0.90 (−2.61, 0.81)	−0.61 (−3.25, 2.04)	–

Bold values indicate statistically significant differences (*P* < .05) when compared with the control.

CON = control, CR = cognitive rehabilitation, CT = cognitive training, HE = health education, MI = multidomain intervention, MMSE = Mini-Mental State Examination, MT = musical therapy, NPTs = non-pharmacological therapies, PE = physical exercise.

**Figure 4.**
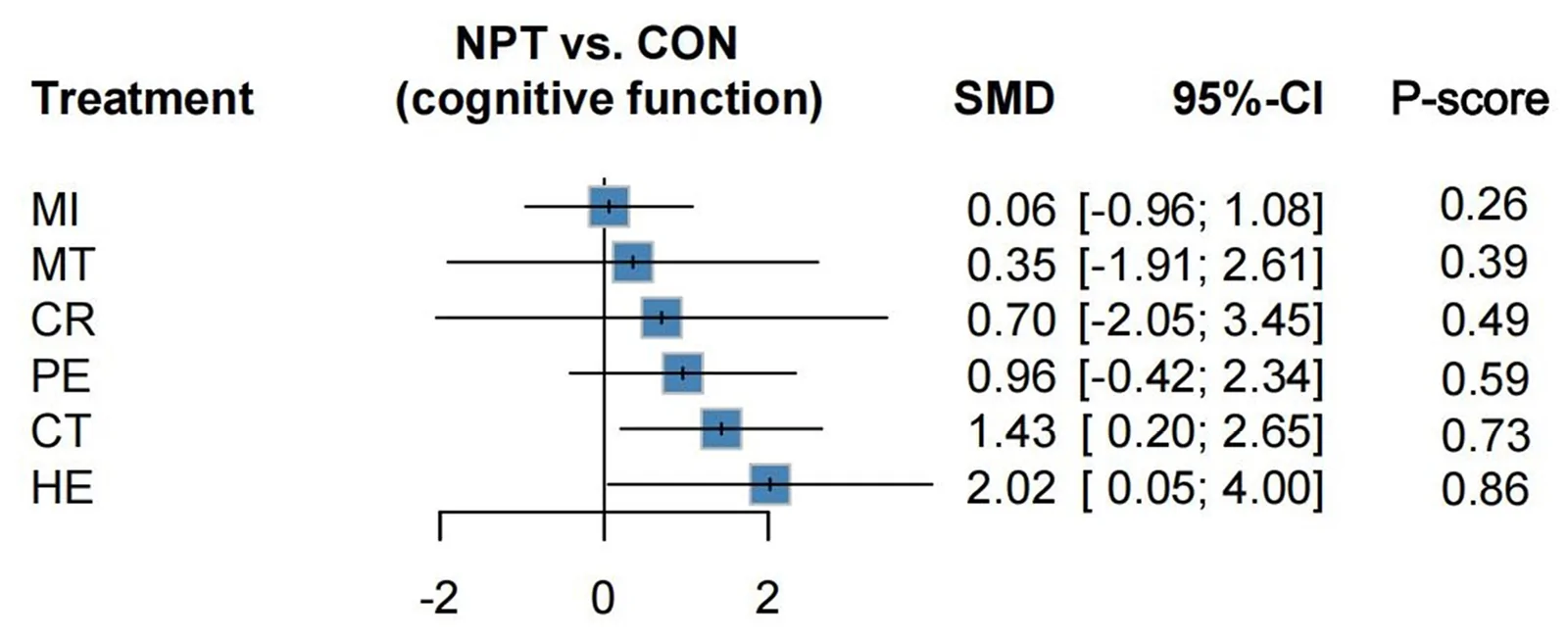
A forest plot depicting the influence of NPTs on cognitive function (MMSE). CI = confidence interval, CON = control, CR = cognitive rehabilitation, CT = cognitive training, HE = health education, MI = multidomain intervention, MMSE = Mini-Mental State Examination, MT = music therapy, NPTs = non-pharmacological therapies, PE = physical exercise, SMD = standardized mean difference.

The outcomes of the subgroup analyses were presented in Table S2, Supplemental Digital Content 6. The results of the subgroup analyses categorized by region and year of publication showed that the Americas and others group (SMD = 0.42, 95% CI = −1.44 to 2.28) and the ≤2018 group (SMD = 0.35, 95% CI = −0.01 to 0.71) of the RCTs produced null effect estimates.

#### 3.3.3. MoCA scores

The NMA of the included studies yielded an *I*^2^ value of 77.6%. The symmetrical distribution of the funnel plot suggested the absence of significant publication bias in this study (Fig. S3, Supplemental Digital Content 7). The coherence between different evidence sources was rigorously examined through comprehensive statistical testing. Global assessment of inconsistency yielded a *P* value of .4082, while pairwise node-splitting analyses produced the following results: CT versus CON (*P* = .3379), PE versus CON (*P* = .8157), HE versus CON (*P* = .2984), CT versus HE (*P* = .2984), and CT versus PE (*P* = .8157). These results support the validity of the consistency model in our NMA (Table S5, Supplemental Digital Content 8 and Fig. S4, Supplemental Digital Content 9).

The study results indicated that PE (SMD = 2.26, 95% CI = 0.95–3.57), CT (SMD = 2.96, 95% CI = 1.57–4.35), and HE (SMD = 2.43, 95% CI = 0.43–4.43) demonstrated statistically significant superiority in efficacy compared to the control group. In contrast, MI (SMD = −1.00, 95% CI = −3.52 to 1.52) and MT (SMD = 1.21, 95% CI = −0.73 to 3.15) failed to demonstrate significant therapeutic effects. The exploratory P-scores for PE, HE, and CT were 0.69, 0.73, and 0.89, respectively (Table 3 and Fig. 5).

**Table 3:** The influence of NPTs on cognitive performance was determined through standardized cognitive assessments (MoCA).

Group	CON	CT	HE	MI	MT	PE
CON	–	–	–	–	–	–
CT	**−2.96 (−4.35, −1.57**)	–	–	–	–	–
HE	**−2.43 (−4.43, −0.43**)	0.53 (**−**1.40, 2.45)	–	–	–	–
MI	1.00 (**−**1.52, 3.52)	3.96 (1.08, 6.84)	3.43 (0.21, 6.65)	–	–	–
MT	**−**1.21 (**−**3.15, 0.73)	1.75 (**−**0.64, 4.13)	1.22 (**−**1.56, 4.01)	**−**2.21 (**−**5.39, 0.97)	–	–
PE	**−2.26 (−3.57, −0.95**)	0.70 (**−**0.97, 2.37)	0.17 (**−**2.11, 2.46)	**−**3.26 (**−**6.11, −0.41)	**−**1.05 (**−**3.39, 1.29)	–

Bold values indicate statistically significant differences (*P* < .05) when compared with the control.

CON = control, CT = cognitive training, HE = health education, MI = multidomain interventions, MoCA = The Montreal Cognitive Assessment, MT = musical therapy, NPTs = non-pharmacological therapies, PE = physical exercise.

**Figure 5.**
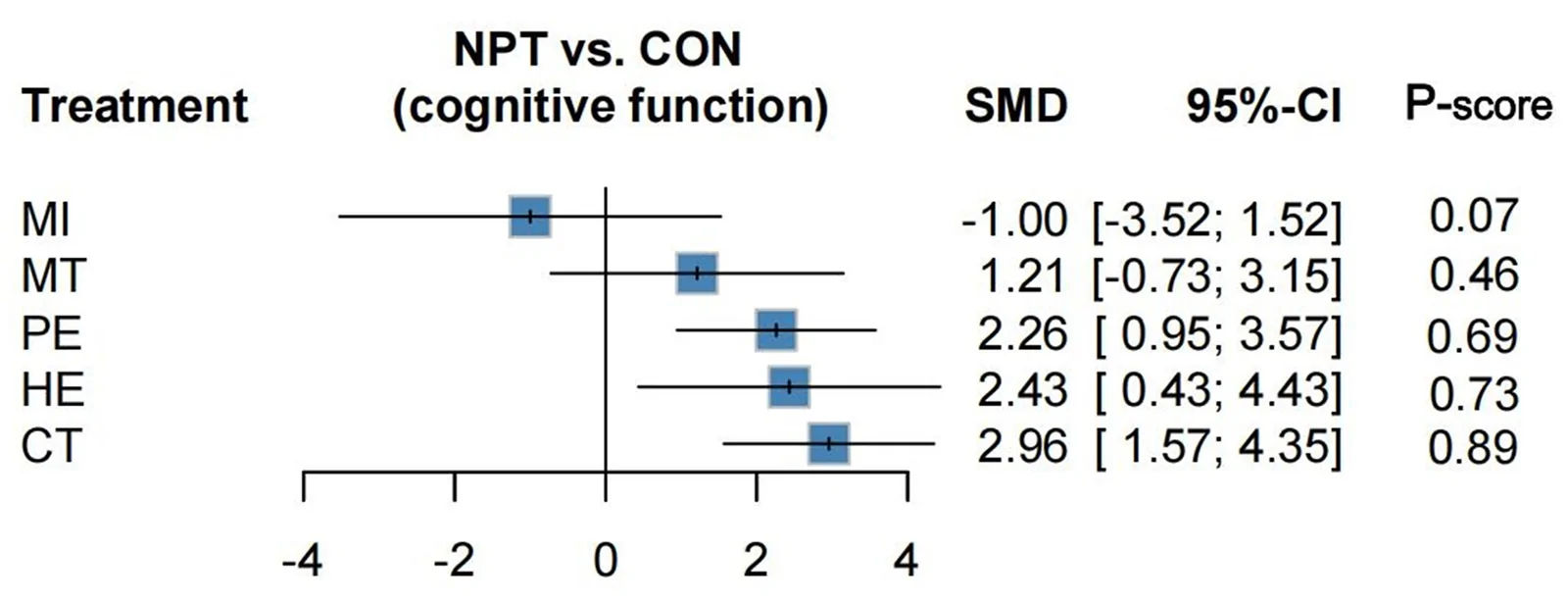
A forest plot depicting the influence of NPTs on cognitive function (MoCA). CI = confidence interval, CON = control, CT = cognitive training, HE = health education, MI = multidomain intervention, MoCA = Montreal Cognitive Assessment, MT = music therapy, NPTs = non-pharmacological therapies, PE = physical exercise, SMD = standardized mean difference.

The results of the subgroup analyses were displayed in Table S3, Supplemental Digital Content 10. The findings of the subgroup analyses, categorized by region and year of publication, revealed an absence of effect for RCTs in the Europe group (SMD = 0.31, 95% CI = −0.06 to 0.69) and in the ≥2022 group (SMD = 0.41, 95% CI = −0.04 to 0.86).

## 4. Discussion

Numerous studies have demonstrated the effectiveness of NPTs in enhancing cognitive function in patients with dementia,^[39–42]^ but the most effective strategy remains unclear. To address this, we conducted a NMA comparing various NPTs. Our results showed that CT and HE significantly improved MMSE scores, while PE, HE, and CT significantly improved MoCA scores compared to control. Despite these limitations, our findings provide valuable comparative evidence to inform the selection of NPTs in clinical practice and to guide the design of future head-to-head trials.

The MMSE and MoCA are widely used cognitive assessment tools. The MMSE is particularly sensitive to memory and language domains, whereas the MoCA has higher sensitivity and specificity for detecting MCI. Different interventions may affect different cognitive domains differently, suggesting that intervention strategies should be selected based on individual patient profiles.

The term CT involves structured cognitive exercises aimed at maintaining or improving cognitive performance. Initially delivered in paper-and-pencil format,^[43–45]^ CT has increasingly shifted to computerized platforms.^[15]^ The superior efficacy of CT observed in our study aligns with previous reports,^[46,47]^ and may be attributed to its multiple mechanisms: promoting neuroplasticity, enhancing cognitive reserve, and enabling adaptive training through computerized protocols. Among the 6 NPTs examined, CR did not show significant efficacy. This is consistent with the view that CR is primarily a compensatory intervention aimed at maintaining daily functioning rather than enhancing cognitive function per se,^[48]^ a distinction supported by a scoping review.^[49]^ However, the potential benefit of CR for MCI cannot be ruled out, given the absence of large-scale, high-quality RCTs; further studies are needed to clarify its effects.

PE and HE are increasingly used as preferred NPTs for cognitive disorders due to their safety and cost-effectiveness, with multiple RCTs confirming their cognitive benefits.^[50–52]^ PE includes aerobic, resistance, and mind-body exercises, with effects potentially varying by exercise type, intensity, frequency, and duration. Evidence on optimal exercise regimens remains mixed: some studies favor multimodal programs,^[53,54]^ while others suggest resistance training is superior,^[55]^ indicating that further optimization of exercise protocols is warranted. HE, defined as planned educational activities to promote healthy behaviors, has also shown efficacy in improving cognitive function in MCI patients.^[32,56,57]^ HE has been associated with broader benefits including improved functionality and quality of life^[58]^; the present study’s findings aligned with this argument. Although various HE formats exist, their relative effectiveness remains unclear and warrants further investigation.

MI was defined as a combination of at least 2 NPTs. MT included active participation (e.g., singing, playing instruments) or receptive engagement (e.g., listening, music-assisted relaxation) with music, which utilizes structured musical elements including rhythm, tempo, pitch, melody, and harmony to achieve therapeutic goals.^[59–61]^ Neither MI nor MT showed statistically significant effects compared to control, which is contrary to some prior reports. This discrepancy may be attributed to the limited number and methodological quality of included studies – only 3 MT trials met our criteria, versus 7 for CT – as well as heterogeneity in MT regimens (active vs receptive) and intervention duration. Furthermore, although 2 of the 3 MT studies used the more sensitive MoCA assessment, no significant effect was detected, suggesting that differences in MT protocols rather than assessment tool sensitivity may account for this result. Notably, MT operates through mechanisms distinct from CT, such as rhythmic and improvisational processes that may not be fully captured by conventional cognitive tests, and requires more tailored assessment methods and more adequately designed intervention cycles to accurately assess their clinical effects. To ensure the scientific rigor of the study, further confirmation was necessary.

Available studies suggest that NPTs might enhance cognitive performance through multi-targeted mechanisms: enhanced production of neurotrophic factors coupled with volumetric increases in prefrontal and anterior cingulate cortical regions^[62]^; the modulation of inflammation-associated cytokine levels,^[63,64]^ attenuation of oxidative stress damage^[65]^; and enhancement of cerebral blood flow perfusion,^[66]^ and inhibition of hyperphosphorylation of tau proteins to slow the dementia process.^[67,68]^ However, most of this evidence derives from animal models, and further validation in humans is needed to confirm the generalizability of these pathways.

This NMA evaluated a range of NPTs for cognitive impairment, incorporating more RCTs and greater intervention diversity than prior syntheses, and enabling integrated direct and indirect comparisons. In addition, we included both MMSE and MoCA as outcome measures, overcoming the limitation of traditional studies using only MMSE. However, the present study was not without its limitations. First, despite the optimal performance of PE, CT, and HE in improving cognitive function in patients with MCI or dementia, few included studies incorporated both methods, and sample sizes were typically small. This may have resulted in an overestimation of the intervention’s effect. Second, most included RCTs did not report the timing of cognitive assessments or follow-up MMSE/MoCA scores, precluding assessment of the intervention effect’s persistence. Third, despite rigorous evaluation of NMA assumptions (homogeneity, transitivity, and consistency), significant between-study heterogeneity persisted, and we did not examine pairwise heterogeneity due to the sparse network structure. Future studies with more direct comparisons between interventions could provide more granular heterogeneity assessments. Fourth, our stringent RCT criteria may have excluded innovative MT approaches published in other formats, and alternative study designs could be considered for such complex interventions. Finally, due to the limited data on the side effects of cognitive interventions in the included studies, this study did not conduct a systematic analysis of their safety, and our study included both MCI and dementia patients. The limited number of dementia-only trials precluded separate network analysis, so the rankings should be applied to dementia patients with caution. The exploratory P-score rankings from NMA cannot be regarded as confirmed clinical efficacy grades.

## 5. Conclusion

Based on the current research evidence, the results of this NMA suggest that PE, CT, and HE demonstrate relatively more significant intervention effects in improving cognitive function in patients with MCI or dementia. Given MT’s unique intervention mechanism and the applicability of existing assessment tools, this study did not find that MT showed statistically significant cognitive improvement effects. In addition, the effect size of MI also did not reach statistical significance. These findings may be used to guide the selection of different NPTs in clinical practice.

## Author contributions

**Data curation:** Hongxia Zhou, Ting Ma.

**Investigation:** Hongxia Zhou, Lingling Yang.

**Conceptualization:** Ting Ma, Hongxia Zhou.

**Methodology:** Ting Ma, Xian Sun.

**Supervision:** Xian Sun, Jiafei Yang.

**Writing – review & editing:** Hongxia Zhou, Ting Ma, Jiafei Yang.






















