# Supplementary material for: Comparative efficacy of various non-pharmacological therapies on cognitive function in patients with mild cognitive impairment or dementia: A network meta-analysis
Source: Medicine (Baltimore). 2026 Sep 25;105(39):e50854. doi: 10.1097/MD.0000000000050854 (PMC13619304; doi:10.1097/MD.0000000000050854)
Supplement: Supplementary file 1 [file medi-105-e50854-s001.docx]

**Table S1 Search strategy for Pubmed**

| #1 | "Cognitive Dysfunction"[MeSH Terms] |
| --- | --- |
| #2 | ((((((((((((((((((((((((Cognitive Dysfunctions[Title/Abstract]) OR (Dysfunction, Cognitive[Title/Abstract])) OR (Dysfunctions, Cognitive[Title/Abstract])) OR (Cognitive Disorder[Title/Abstract])) OR (Cognitive Disorders[Title/Abstract])) OR (Disorder, Cognitive[Title/Abstract])) OR (Disorders, Cognitive[Title/Abstract])) OR (Cognitive Impairments[Title/Abstract])) OR (Cognitive Impairment[Title/Abstract])) OR (Impairment, Cognitive[Title/Abstract])) OR (Impairments, Cognitive[Title/Abstract])) OR (Mild Cognitive Impairment[Title/Abstract])) OR (Cognitive Impairment, Mild[Title/Abstract])) OR (Cognitive Impairments, Mild[Title/Abstract])) OR (Impairment, Mild Cognitive[Title/Abstract])) OR (Impairments, Mild Cognitive[Title/Abstract])) OR (Mild Cognitive Impairments[Title/Abstract])) OR (Cognitive Decline[Title/Abstract])) OR (Cognitive Declines[Title/Abstract])) OR (Decline, Cognitive[Title/Abstract])) OR (Declines, Cognitive[Title/Abstract])) OR (Mental Deterioration[Title/Abstract])) OR (Deterioration, Mental[Title/Abstract])) OR (Deteriorations, Mental[Title/Abstract])) OR (Mental Deteriorations[Title/Abstract]) |
| #3 | "Dementia"[MeSH Terms] |
| #4 | (((((((((((Dementias[Title/Abstract]) OR (Amentia[Title/Abstract])) OR (Amentias[Title/Abstract])) OR (Senile Paranoid Dementia[Title/Abstract])) OR (Dementias, Senile Paranoid[Title/Abstract])) OR (Paranoid Dementia, Senile[Title/Abstract])) OR (Paranoid Dementias, Senile[Title/Abstract])) OR (Senile Paranoid Dementias[Title/Abstract])) OR (Familial Dementia[Title/Abstract])) OR (Dementia, Familial[Title/Abstract])) OR (Dementias, Familial[Title/Abstract])) OR (Familial Dementias[Title/Abstract]) |
| #5 | randomized controlled trial[Publication Type] OR randomized[Title/Abstract] OR placebo[Title/Abstract] |
| #6 | (#1 OR #2) OR (#3 OR #4) AND #5 |
