# Supplementary material for: Comparative efficacy of various non-pharmacological therapies on cognitive function in patients with mild cognitive impairment or dementia: A network meta-analysis
Source: Medicine (Baltimore). 2026 Sep 25;105(39):e50854. doi: 10.1097/MD.0000000000050854 (PMC13619304; doi:10.1097/MD.0000000000050854)
Supplement: Supplementary file 2 [file medi-105-e50854-s003.docx]

**Table S4 MMSE consistency assessment results**

| Comparison | Direct SMD (95% CI) | Indirect SMD (95% CI) | p-value |
| --- | --- | --- | --- |
| CT vs CON | 1.12 (-0.18, 2.42) | 4.05 (0.27, 7.83) | 0.1504 |
| HE vs CON | 3.70 (0.68, 6.72) | 0.77 (-1.84, 3.38) | 0.1500 |
| CT vs HE | 0.35 (-1.92, 2.62) | -2.58 (-5.87, 0.71) | 0.104 |

Abbreviations: CT, cognitive training; HE, health education; CON, control; SMD, standardized mean difference; CI, confidence interval. Consistency analysis using node splitting method.
