# Supplementary material for: Comparative efficacy of various non-pharmacological therapies on cognitive function in patients with mild cognitive impairment or dementia: A network meta-analysis
Source: Medicine (Baltimore). 2026 Sep 25;105(39):e50854. doi: 10.1097/MD.0000000000050854 (PMC13619304; doi:10.1097/MD.0000000000050854)
Supplement: Supplementary file 3 [file medi-105-e50854-s006.docx]

**Table S2 Subgroup Analysis Results of MMSE**

| Subgroup | | | patients | | SMD 90%CI | Heterogeneity  (I^2^) | P-value |
| --- | --- | --- | --- | --- | --- | --- | --- |
|  |  |  | Exp | CON |  |  |  |
| MMSE | Region | East Asia | 323 | 363 | 0.39 (0.09, 0.68) | 70% | 0.0010 |
|  |  | Europe | 533 | 399 | 0.36 (0.09, 0.64) | 68% | 0.0090 |
|  |  | Americas and Others | 30 | 32 | 0.42 (-1.44, 2.28) | 91% | 0.0003 |
|  | Publication years | Years ＞ 2018 | 383 | 413 | 0.39 (0.15, 0.63) | 63% | 0.0020 |
|  |  | Years ≤ 2018 | 503 | 381 | 0.35 (-0.01, 0.71) | 77% | 0.0500 |
