# Supplementary material for: Comparative efficacy of various non-pharmacological therapies on cognitive function in patients with mild cognitive impairment or dementia: A network meta-analysis
Source: Medicine (Baltimore). 2026 Sep 25;105(39):e50854. doi: 10.1097/MD.0000000000050854 (PMC13619304; doi:10.1097/MD.0000000000050854)
Supplement: Supplementary file 4 [file medi-105-e50854-s008.docx]

**Table S5 MoCA consistency assessment results**

| Comparison | Direct SMD (95% CI) | Indirect SMD (95% CI) | p-value |
| --- | --- | --- | --- |
| CT vs CON | 2.46 (0.74, 4.18) | 3.89 (1.53, 6.25) | 0.3379 |
| HE vs CON | 3.48 (0.67, 6.29) | 1.36 (-1.48, 4.20) | 0.8157 |
| PE vs CON | 2.33 (0.88, 3.78) | 1.92 (-1.20, 5.04) | 0.2984 |
| CT vs HE | 1.30 (-1.12, 3.72) | -0.82 (-4.01, 2.36) | 0.2984 |
| CT vs PE | 0.95 (-1.75, 3.65) | 0.54 (-1.59, 2.67) | 0.8157 |

Abbreviations: CT, cognitive training; HE, health education; PE, physical exercise; CON, control; SMD, standardized mean difference; CI, confidence interval. Consistency analysis using node splitting method.
