# Supplementary material for: Comparative efficacy of various non-pharmacological therapies on cognitive function in patients with mild cognitive impairment or dementia: A network meta-analysis
Source: Medicine (Baltimore). 2026 Sep 25;105(39):e50854. doi: 10.1097/MD.0000000000050854 (PMC13619304; doi:10.1097/MD.0000000000050854)
Supplement: Supplementary file 5 [file medi-105-e50854-s010.docx]

**Table S3 Subgroup Analysis Results of MoCA**

| Subgroup | | | patients | | SMD 90%CI | Heterogeneity  (I^2^) | P-value |
| --- | --- | --- | --- | --- | --- | --- | --- |
|  |  |  | Exp | CON |  |  |  |
| MoCA | Region | Asia | 317 | 361 | 1.02 (0.40, 1.64) | 93% | 0.0010 |
|  |  | Europe | 404 | 441 | 0.31 (-0.06, 0.69) | 32% | 0.1000 |
|  | Publication years | Years ≥ 2022 | 220 | 257 | 0.41 (-0.04, 0.86) | 82% | 0.0800 |
|  |  | Years ＜ 2022 | 184 | 184 | 1.34 (0.47, 2.20) | 92% | 0.003 |
